# Stroke Mimicking Symptoms and Consequences of Alcohol Intoxication: A Case Report

**DOI:** 10.7759/cureus.62305

**Published:** 2024-06-13

**Authors:** Mauer Gonçalves, Maria Elena Lopez, Claudia Di Bella, Humberto Morais

**Affiliations:** 1 Medicine, Centro de Estudos Avançados em Educação e Formação Médica, Agostinho Neto University, Luanda, AGO; 2 Cardiology, Luanda Medical Center, Luanda, AGO; 3 Pediatric Neurology, Luanda Medical Center, Luanda, AGO; 4 Internal Medicine, Luanda Medical Center, Luanda, AGO; 5 Cardiology, Hospital Militar Principal/Instituto Superior, Luanda, AGO

**Keywords:** thalamic ischemic stroke, car accident, ataxia, alcoholic intoxication, hypertension

## Abstract

A 41-year-old Black male with a history of hypertension was involved in a car accident, after which he exhibited symptoms such as slow and incoherent speech, unstable gait, dizziness, drowsiness, slow thinking, and loss of strength in his limbs. Despite multiple negative alcohol tests, his symptoms mimicked those of acute alcohol intoxication. Upon presentation to the emergency room, physical examination and brain imaging revealed a right anterior thalamic ischemic infarction. He was discharged completely recovered after two days without sequelae. This case underscores the importance of considering stroke as a differential diagnosis in patients presenting with symptoms similar to alcohol intoxication, particularly in hypertensive individuals.

## Introduction

Sub-Saharan Africa has considerable deficits in epidemiological records; however, an increase in deaths from cardiovascular events has been estimated in the region. It is known that Africa suffers the greatest impact worldwide from arterial hypertension, which is the most important risk factor for the presence of stroke. In the literature, the incidence of cerebral vascular events on the continent is located at 316 per 100,000 inhabitants and the prevalence at 981 cases per 100,000 inhabitants, which constitutes a public health problem [1,2].

African countries, like other nations with a low development index, face an increasing incidence of stroke. In these regions, 86% of stroke-related fatalities occur, in contrast to a decrease in such cases in developed countries [3].

According to a descriptive study conducted in a tertiary health center in Angola, the most affected group was men over 55 years of age. In this study series, the presence of hypertension was the main risk factor, followed by heart disease and diabetes mellitus [4].

Strokes can happen at any time during various daily activities. People collectively spend millions of hours behind the wheel each year. It is quite likely that some drivers experience a stroke while on the road, and unfortunately, a portion of them may contribute to car accidents. Research conducted in Japan indicates that 4% of all strokes occur while driving, with accidents arising in 16% of these cases [5,6].

Furthermore, stroke can manifest with clinical features similar to acute alcohol intoxication, characterized by mood swings, disinhibition, slow thinking, reduced attention and concentration, incoordination, slurred speech, and an unsteady gait [7,8].

A thalamic stroke occurs due to the disruption of small vessels arising from various arteries at the cranial base, with the posterior communicating artery, the internal carotid artery, and the posterior cerebral artery being the primary vascular elements involved. Additionally, in a published case series of patients with stroke, the proportion of ischemic thalamic attacks in hypertensive young adult men should not be underestimated [9].

The intricate interplay between the nucleus and corticothalamic and thalamocortical pathways, along with the connections among its various subnuclear components, results in a wide range of clinical manifestations when lesions occur at this level. These manifestations include motor symptoms such as hemiparesis and ataxia, variations in higher psychofunctional processes like attention, motivation, and alertness, alterations in behavior, memory, and language, as well as other cognitive and emotional dysfunctions. Additionally, lesions in the thalamic, subcortical white matter and brainstem areas may exhibit clinical expressions that are similar to those seen in cerebellar involvement [10,11].

This paper describes the evolution of a known hypertensive patient who was admitted to the emergency room complaining of dizziness, drowsiness, slow thinking, loss of strength in the limbs, and ataxia and who had a car accident. Brain imaging showed features compatible with a recent ischemic infarction involving the anterior thalamic region and the adjacent thalamic-diencephalic transition on the right.

From the research conducted, we did not find any similar cases in sub-Saharan African populations in the literature. The interest in this case lies in the fact that it is a rare condition with an unusual presentation in a relatively young individual.

## Case presentation

A 41-year-old Black male presented to the emergency room after crashing his car into the back of another vehicle. He displayed slow and incoherent speech along with an unstable gait, prompting police officers to conduct four alcohol tests, all of which yielded negative results. The following day, he sought medical attention at the Luanda Medical Center, reporting symptoms that had developed over 48 hours, including a sensation of drunkenness despite not consuming alcohol, dizziness, drowsiness, slowed thinking, weakness in the limbs, and an ataxic gait. He denied experiencing a fever and headache.

The patient had been diagnosed with hypertension four years prior and was being treated with perindopril and amlodipine at a dose of 10/10 mg/day. He had a medical history of bilateral hydronephrosis and bilateral renal lithiasis, for which he had undergone three ureteroscopies. He reported light alcohol consumption but denied smoking.

During the physical examination, the patient was alert and oriented, exhibiting dysarthria and an ataxic gait. Osteotendinous reflexes were rated as II and IV in the right hemibody. Vital signs were within normal limits, with blood pressure measuring 140/89 mmHg, heart rate at 63 bpm, and SpO2 at 98%. Pulmonary and cardiac examinations revealed no abnormalities.

**Figure 1:**
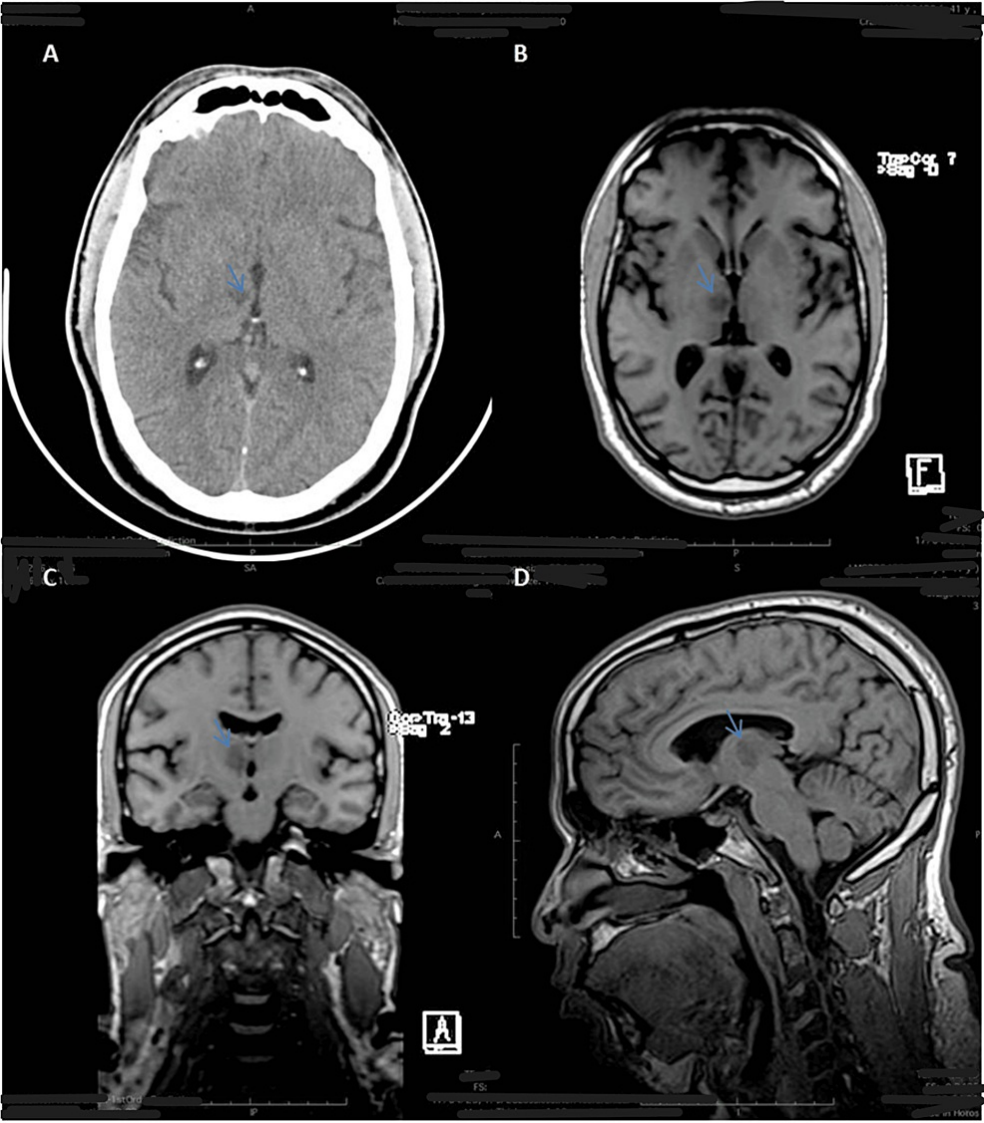
(A) Cranioencephalic CT scan revealing a right anterior thalamic ischemic infarction (blue arrow). (B, C, and D) Cerebral MRI illustrating findings consistent with a recent ischemic infarction, encompassing the anterior thalamic region and the adjacent thalamodiencephalic transition on the right (blue arrow)

The patient’s resting ECG revealed sinus rhythm with a heart rate of 65 bpm and no significant changes. Echocardiography conducted upon admission showed concentric left ventricular hypertrophy with preserved global systolic function (EF = 57%, LGS = 16.4%). Laboratory results upon admission, as shown in Table 1, indicated elevated cholesterol and triglyceride levels. A cranioencephalic CT scan revealed an ischemic infarction in the right anterior thalamic region (Figure 1A). Cerebral MRI findings were consistent with a recent ischemic infarction involving the anterior thalamic region and the adjacent thalamodiencephalic transition on the right, within the perforating vertebral basilar territory, with no significant mass effect or signs of hemorrhagic transformation (Figure 1B, 1C, 1D).

**Table 1:** Laboratory values upon admission and corresponding normal ranges HDL, high-density lipoprotein; LDL, low-density lipoprotein

Laboratory parameters	Upon admission	Normal range
Hemoglobin	14.8	12-16 g/dL
Total cholesterol	241 mg/dL	<200 mg/dL
Triglycerides	151 mg/dL	<150 mg/dL
HDL cholesterol	31 mg/dL	>40 mg/dL
LDL cholesterol	180 mg/dL	<120 mg/dL
Creatinine	1.1 mg/dL	0.8-1.3 mg/dL
Urea	13 mg/dL	20-50 mg/dL
Sodium	140 mmol/L	135-145 mmol/L
Potassium	4.3 mmol/L	3.4-4.5 mmol/L
Chloride	105 mmol/L	95-108 mmol/L

During hospitalization, the patient was medicated with perindopril + amlodipine 10/10 mg/day, atorvastatin 20 mg/day, and aspirin 100 mg/day. After two days of hospitalization, he was discharged completely recovered, without sequelae, and was prescribed the same medication.

## Discussion

Occasionally, the literature reports instances of acute cardiovascular events happening while driving [5,6,12,13]. Most of these studies were carried out by forensic scientists or accident investigators, focusing on patients whose vascular events were severe enough to lead to accidents [12,13]. Previous studies have reported that about 4% of acute strokes are driving related, with ischemic strokes more likely to occur while driving compared to hemorrhagic strokes [5,6].

The case report aligns with the trends observed in the literature, emphasizing the impact of stroke on men over 55 years old, with hypertension being the predominant risk factor [14]. This corroborates findings from a descriptive study conducted in Angola, pointing toward a consistent pattern in the region [4].

One noteworthy aspect is the association between strokes and automobile accidents. The case of a known hypertensive patient experiencing a stroke while driving, resulting in a car accident, underscores the potential dangers and consequences. This scenario calls for increased awareness and strategies to manage individuals with preexisting health conditions that may lead to sudden incapacitation, impacting their ability to operate vehicles safely [5,6,13].

The thalamus is a part of the diencephalon, containing numerous connections between the forebrain and subcortical structures [11,12]. It regulates several functions, including sensation, language, speech, mood, and behavior [15].

A thalamic stroke occurs when there is a disruption in blood flow to the thalamus. After a stroke, there may be short- or long-term effects on sensation, language, mood, or motor function. Similar to other strokes, some risk factors for thalamic strokes and infarcts are high blood pressure, obesity, sedentarism, alcohol, diet, obesity, smoking, stress, drug use like cocaine and methamphetamine, high cholesterol, diabetes, sleep apnea, cardiovascular disease, heart failure, heart defects, heart infection, abnormal heart rhythms, family history of strokes, heart attacks, and COVID-19 [16-18].

In addition to those risk factors, specific demographics of people are more likely to get strokes than others. These demographics are people over the age of 55, African American people, men, and people who use birth control or hormone therapy [15,17].

After a thalamic stroke, people may experience the following side effects: speech and language difficulties. The thalamus is involved in several cognitive and motor functions, including speech. After a thalamic stroke, a person may lose the ability to understand language and speech. Sometimes this is temporary and gets better. Behavioral and mood changes are common after a thalamic stroke due to the role of the thalamus in regulating mood and motivation [15,17].

The clinical presentation of stroke can be diverse, as illustrated in this case, where the patient exhibited symptoms mimicking acute alcohol intoxication. This complexity emphasizes the need for healthcare professionals to consider various manifestations of stroke, especially when patients present with atypical symptoms [7,15].

The detailed case report elucidates the evolution of a hypertensive patient who, despite negative alcohol tests, displayed symptoms consistent with alcohol intoxication. The imaging studies revealed an ischemic infarction in the anterior thalamic region, highlighting the importance of timely diagnostic procedures in stroke management.

Patients seeking treatment after more than 24 hours from the onset of symptoms are usually not candidates for standard thrombolysis. This is because over time, the potential benefits of thrombolysis decrease, and the risks of complications, such as bleeding, become more prominent [19].

The patient’s swift recovery without sequelae after receiving medical intervention further underscores the significance of prompt and effective treatment in mitigating the consequences of stroke.

## Conclusions

This case report highlights the importance of early recognition of symptoms resembling alcohol intoxication as possible indicators of stroke, especially in populations with a high prevalence of arterial hypertension. The atypical presentation, such as dizziness, drowsiness, slow thinking, loss of strength in the limbs, and ataxic gait, can be mistaken for alcohol intoxication, delaying proper diagnosis and treatment. Prompt medical intervention, as demonstrated in this case, is crucial for recovery without sequelae. This report underscores the need for greater awareness and training among healthcare professionals to identify and appropriately manage stroke symptoms, regardless of the patient’s age.
